# Novel Scaffold Agonists of the α_2A_ Adrenergic Receptor Identified via Ensemble-Based Strategy

**DOI:** 10.3390/molecules29051097

**Published:** 2024-02-29

**Authors:** Shiyang Sun, Pengyun Li, Jiaqi Wang, Dongsheng Zhao, Tingting Yang, Peilan Zhou, Ruibin Su, Zhibing Zheng, Song Li

**Affiliations:** 1 National Engineering Research Center for Strategic Drugs, Beijing Institute of Pharmacology and Toxicology, Beijing 100850, China; noah97sun@163.com (S.S.); bnuhuaxuelpy@163.com (P.L.); lis@bmi.ac.cn (S.L.); 2 State Key Laboratory of Toxicology and Medical Countermeasures, Beijing Institute of Pharmacology and Toxicology, Beijing 100850, China; 18645278089@163.com; 3 Academy of Military Medical Sciences, Beijing 100850, China; dszhao@bmi.ac.cn; 4 State Key Laboratory of Natural and Biomimetic Drugs, School of Pharmaceutical Sciences, Peking University, Beijing 100191, China; ytt_kid@163.com

**Keywords:** α_2A_-AR, ensemble-based screening, molecular dynamics simulation, bitopic agonist

## Abstract

The α_2A_ adrenergic receptor (α_2A_-AR) serves as a critical molecular target for sedatives and analgesics. However, α_2A_-AR ligands with an imidazole ring also interact with an imidazoline receptor as well as other proteins and lead to undesirable effects, motivating us to develop more novel scaffold α_2A_-AR ligands. For this purpose, we employed an ensemble-based ligand discovery strategy, integrating long-term molecular dynamics (MD) simulations and virtual screening, to identify new potential α_2A_-AR agonists with novel scaffold. Our results showed that compounds **SY-15** and **SY-17** exhibited significant biological effects in the preliminary evaluation of protein kinase A (PKA) redistribution assays. They also reduced levels of intracellular cyclic adenosine monophosphate (cAMP) in a dose-dependent manner. Upon treatment of the cells with 100 μM concentrations of **SY-15** and **SY-17**, there was a respective decrease in the intracellular cAMP levels by 63.43% and 53.83%. Subsequent computational analysis was conducted to elucidate the binding interactions of **SY-15** and **SY-17** with the α_2A_-AR. The binding free energies of **SY-15** and **SY-17** calculated by MD simulations were −45.93 and −71.97 kcal/mol. MD simulations also revealed that both compounds act as bitopic agonists, occupying the orthosteric site and a novel exosite of the receptor simultaneously. Our findings of integrative computational and experimental approaches could offer the potential to enhance ligand affinity and selectivity through dual-site occupancy and provide a novel direction for the rational design of sedatives and analgesics.

## 1. Introduction

The α_2_ adrenergic receptors (α_2_-ARs), comprised of α_2A_, α_2B_, and α_2C_-subtypes, are G-protein coupled receptors (GPCRs) expressed throughout the brain, heart, and vasculature, as well as in the kidney and platelets [1]. In the brain, α_2A_-ARs predominate, accounting for approximately 90% of the total α_2_-AR expression, with notable concentrations in the locus coeruleus and the prefrontal cortex. The α_2_-AR agonists act as antihypertensive, sedative, and analgesic drugs through central activation of α_2A_-ARs, with neurological and psychiatric regulation. Once activated by agonists, α_2A_-AR couple to the Gi/o protein to inhibit the activity of adenylyl cyclase, which in turn decreases levels of downstream intracellular cyclic adenosine monophosphate (cAMP), a pivotal secondary messenger [2,3].

**Figure 1:**
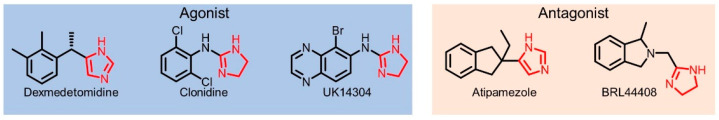
Chemical structures of α_2A_-AR agonists and antagonists.

The ligands of α_2A_-AR are primarily imidazole ring compounds including agonists (dexmedetomidine, clonidine and UK14304) and antagonists (atipamezole and BRL44408) (Figure 1). However, previous studies have shown that the imidazole ring of these ligands also interacts with other proteins, such as the imidazoline receptor and trace amine-associated receptor (TAAR), which cause unavoidable side effects [4,5,6]. These limitations motivate searches for more α_2_-AR ligands with novel scaffolds and binding modes.

As a result of recent breakthroughs in structural biology, an increasing number of GPCRs and ligand complex structures have been identified, including the α_2_-AR with antagonist and agonist [7,8]. Bitopic ligands have also emerged as a promising field for GPCRs, which simultaneously occupy the orthosteric and allosteric sites of the receptor. To date, bitopic ligands have been reported for several class A GPCRs, including the D3 dopamine receptor, µ-opioid receptor, and cannabinoid receptor type 2 (CB2R) [9,10,11]. However, to our knowledge, no bitopic agonists targeting α_2A_-AR have been reported. Therefore, it is a brave and innovative attempt to develop bitopic agonists of α_2A_-AR. In this study, we used an ensemble-based screening strategy, combining MD simulation and molecular docking, to identify two potential novel scaffolds α_2A_-AR agonists. Our findings provide an orientation for the development of novel α_2A_-AR agonists.

## 2. Results and Discussion

### 2.1. Potential Agonists Identified Using the Ensemble-Based Screening Strategy

**Figure 2:**
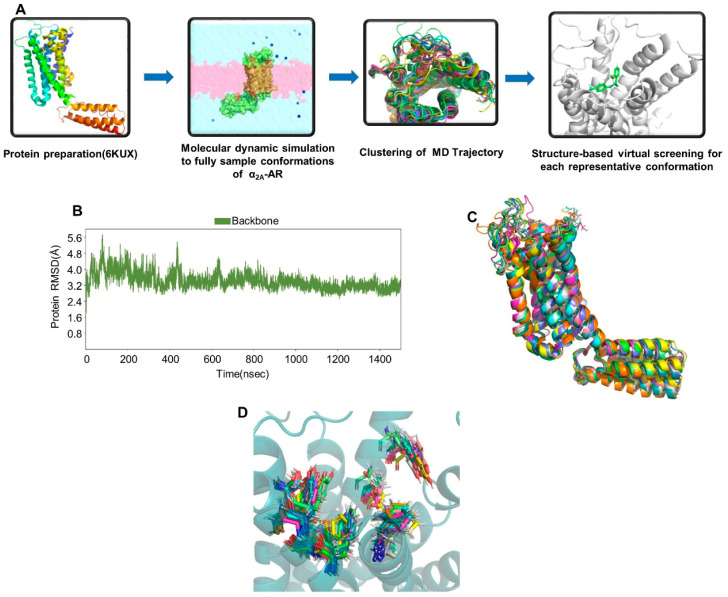
(**A**) Workflow of ensemble-based screening strategy for novel α_2A_-AR agonists; (**B**) RMSD of protein backbone during MD simulation; (**C**) conformations of 10 clusters and crystal structures; (**D**) conformational differences of key binding site residues for 10 clusters.

The receptor-binding sites consist of 10–20 residues with multiple rotatable conformations, which are significantly more than the rotatable torsions of the ligands [12]. Different ligands can interact with the protein in slightly different ways due to the protein’s flexibility. However, the ensemble-based screening strategy that combines MD simulation and molecular docking adequately accounts for the flexibility of protein-binding pockets and aims to enhance the hit rate of virtual screening [13,14]. In this study, we utilized a set of α_2A_-AR conformations for screening instead of a single conformation to replicate the dynamic properties of proteins and reduce bias in our screening. The compound screening workflow is depicted in Figure 2A; firstly, a model of α_2A_-AR in relaxed conformation retrieved from a protein data bank was prepared by maestro (Figure S1). Then MD simulation was conducted to sample the dynamic conformations of the receptor without ligands; representative conformations were extracted by the clustering of the MD trajectory. Finally, virtual screening was performed for each conformation using the Chemdiv library (∼1,600,000 compounds).

To probe the flexibility and dynamic changes of α_2A_-AR at the atomistic level, a 1.5 μs of long-term MD simulation for a receptor without ligands was carried out, calculating the RMSD of protein over the entire 1500 ns trajectory to check whether the simulation system converged or not [15]. As shown in Figure 2B, the system converged after 600 ns of simulation, with an average relatively small RMSD value (about 3.5 Å), suggesting that the protein backbone was stable throughout the trajectory. Next, we identified populated conformations for simulation trajectory by clustering. The simulated trajectory of the protein was divided into ten clusters. Compared with the crystal complex, the conformations of these clusters showed slight differences which can be seen in the receptor and the side chains with different rotamer states (Figure 2C,D).

**Figure 3:**
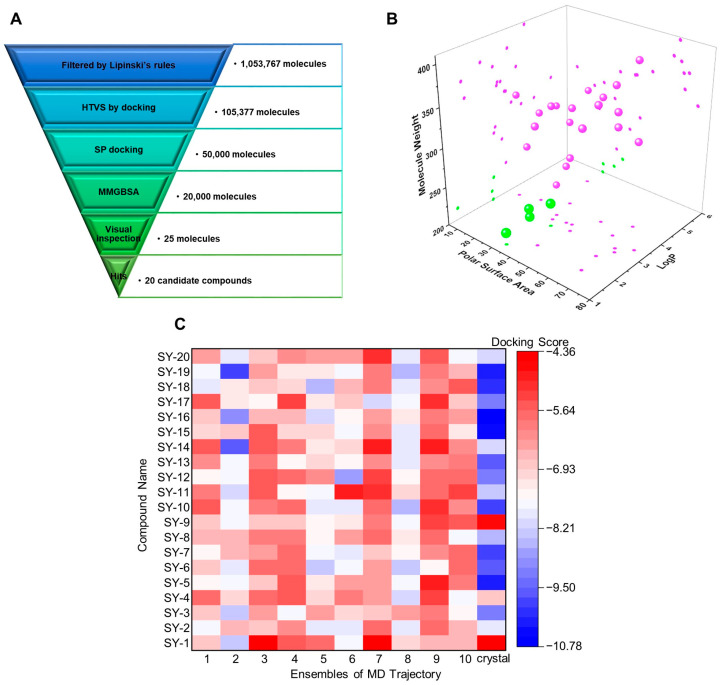
Structure-based virtual screening for novel α_2A_-AR agonists. (**A**) Workflow of virtual screening in Chemdiv library; (**B**) LogP, molecular weight and PSA comparison of candidate compounds (pink) with known α_2A_-AR ligands (green); (**C**) docking scores of 20 molecules for each cluster.

The conformations of 10 clusters and crystal structures were used for structure-based virtual screening. As shown in Figure 3A, Lipinski’s rules, HTVS, SP docking, and MM/GBSA were performed step-by-step to screen potential α_2A_-AR agonists. A total of 1,601,763 compounds from the Chemdiv library were prepared by LigPrep and 1,053,767 of them were filtered by Lipinski’s rules. HTVS and SP docking were then used for each cluster to screen potentially activate molecules. The average docking score for molecules up to a total of 11 conformations was calculated, and the 20,000 top-ranking compounds were selected for binding free energy calculations using the MM/GBSA method and clustering analysis was performed for 2000 of them by 2D fingerprints in maestro. Next, visual inspection including the physicochemical properties of these compounds, the binding free energy, and their binding modes with a receptor as well as structural diversity was conducted to obtain 25 molecules. Finally, 20 of them were currently in stock and finally evaluated for in vitro assay. As shown in Figure 3B and Figure S2, the chemical properties of candidate molecules were full of diverse scaffolds, which were different from the existing ligands of α_2A_-AR. The average docking score and binding free energy demonstrated the high affinity for candidate compounds with α_2A_-AR (Table 1). We also explored the docking scores of 20 molecules for 10 clusters and crystal structures. Most scores ranged from −6 to −8 (Figure 3C), which suggested that those molecules could stably bind to α_2A_-AR. In addition, the ADME/T properties of these compound were calculated by QikProp, and all of them exhibited good blood–brain barrier permeability and lower affinity for hERG (Table S1), indicating their low toxicity, while only one compound slightly violated the Lipinski’s rule of five (QPlogPo/w = 5.648).

### 2.2. The Biological Activities of Candidate Compounds in PKA Redistribution and cAMP Assay

**Figure 4:**
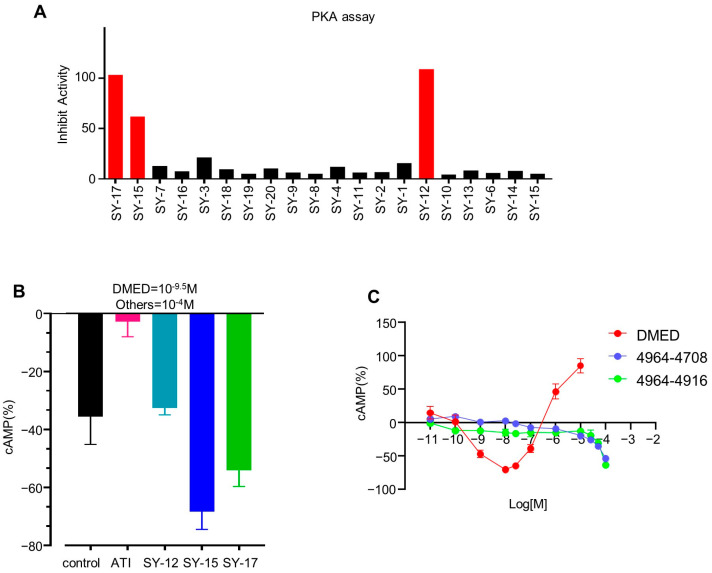
Biological evaluation for candidate compounds based on PKA and cAMP assays. (**A**) Effect of 20 compounds in PKA redistribution assay; (**B**) preliminarily biological evaluation on cAMP assay in vitro for candidate compounds; (**C**) the dose–effect of **SY-17** and **SY-15** in regulating cAMP levels.

Previous studies have shown that activation of α_2A_-ARs can inhibit adenylate cyclase activity to exert physiological effects through the PKA signaling pathway [16,17]. The catalytic domain of PKA, labeled with enhanced green fluorescent protein (PKAcat-EGFP), is typically localized in highly fluorescent aggregates in the cytoplasm for unstimulated cells [18]. Once activated by cAMP, the PKAcat-EGFP fusion protein will redistribute and result in the reduction of fluorescent spots within the cytoplasm. These 20 purchased compounds were next conducted for their effect on the PKA redistribution assay with a single concentration (100 μM). In addition to atipamezole, compounds **SY-12**, **SY-15**, and **SY-17** also showed significant activities to affect the redistribution of PKA within cells (Figure 4A). This result suggested that these three compounds may interact with receptors and change the concentration of intracellular cAMP, thereby influencing the activity of cAMP-dependent PKA, culminating in observable changes in the fluorescence of the PKAcat-EGFP. Three compounds exhibited biological activity in the PKA assay, with a hit rate of 15%, indicating that the ensemble-based screening strategy is effective in identifying potential ligands. In order to further verify whether the compounds **SY-12**, **SY-15**, and **SY-17** affect the redistribution of PKA by acting on α_2A_-AR, we performed an intracellular cAMP assay.

Both Gi/o and Gs can couple to α_2A_-AR while exhibiting opposite effects. The former decreases the levels of intracellular cAMP, while the latter increases them [19]. In the cAMP assay, HEK293 cells expressing α_2A_-AR were treated with DMED (10^−9.5^ M) accompanied with atipamezole, **SY-12**, **SY-15**, or **SY-17** (10^−4^ M) to detect the cAMP levels. We tested this single-concentration cAMP assay to determine whether a candidate molecule was an agonist or antagonist. If the compound reduced the level of intracellular cAMP, it proves that the molecule showed a synergistic effect with DMED, indicating that it was an agonist. Otherwise, it was an antagonist. As shown in Figure 4B, **SY-17** and **SY-15** significantly decreased the cAMP levels compared to cells treated with DMED alone. Notably, **SY-15** enhanced the potency of DMED by 1.92-fold. These results demonstrated that **SY-15** and **SY-17** exhibited a synergistic effect with DMED by enhancing the coupling between α_2A_-AR and Gi/o. Although **SY-12** showed a significant effect to affect the redistribution of PKA within cells, there was no effect on cAMP level. We considered the reason for this phenomenon may be due to the nonspecific effect of **SY-12**. When α_2A_-AR is activated, intracellular cAMP will be decreased, and cAMP will further affect the redistribution of PKA. Therefore, PKA is more in the downstream signaling pathway than cAMP and may be subject to more non-specific effects. Therefore, we focused more on the alterations in cAMP caused by compounds which were directly regulated by receptors. So, we used the cAMP assay to filter out the molecules that showed the significant effect on a PKA assay but were not acting on α_2A_-AR.

Furthermore, we observed that **SY-17** and **SY-15** decreased intracellular cAMP levels in a dose-dependent manner (Figure 4C). These biological experiments confirmed that the compounds **SY-15** and **SY-17** are potential α_2A_-AR agonists with novel scaffolds that can be used as leads for subsequent optimization.

### 2.3. SY-15 and SY-17 Act as Bitopic α_2A_-AR Agonists by Occupying the Orthosite and Exosite Simultaneously

**Figure 5:**
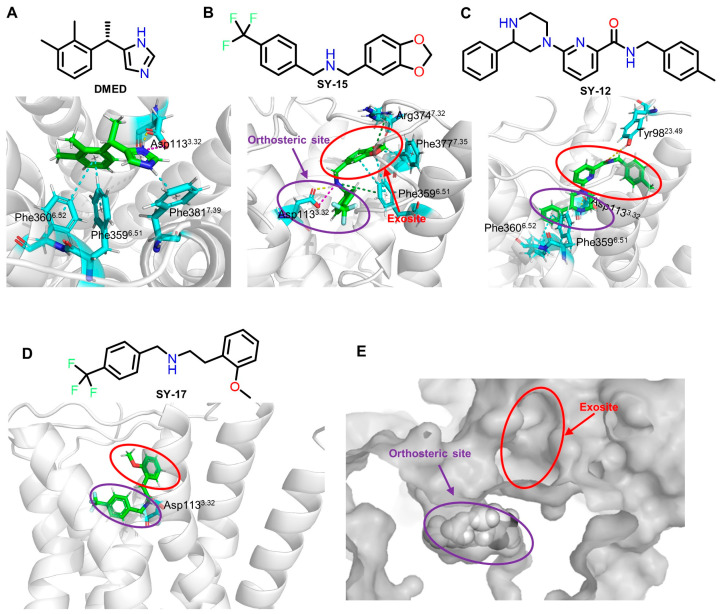
Chemical structures and binding modes of α_2A_-AR ligands. (**A**) Interactions between α_2A_-AR and DMED, with the imidazole ring binding to the orthosteric site with a slat bridge and hydrogen bond; (**B**–**D**) binding mode of molecules **SY-12**, **SY-15**, and **SY-17** with α_2A_-AR; (**E**) exosite and orthosteric site of α_2A_-AR.

Based on the virtual screening and biological experiments previously described, we next investigated the binding modes of these agonists with α_2A_-AR. The molecular docking revealed that DMED, **SY-12**, **SY-15**, and **SY-17** could interact with residues Asp113, a critical determinant for the binding of α_2A_-AR ligands (Figure 5A–D). Intriguingly, partial groups of **SY-12**, **SY-15**, and **SY-17** reached into a novel pocket (exosite) located above the orthosteric site (Figure 5E), suggesting that **SY-12**, **SY-15**, and **SY-17** might act as bitopic agonists, which have not been reported in previous studies for α_2A_-AR ligands.

**Figure 6:**
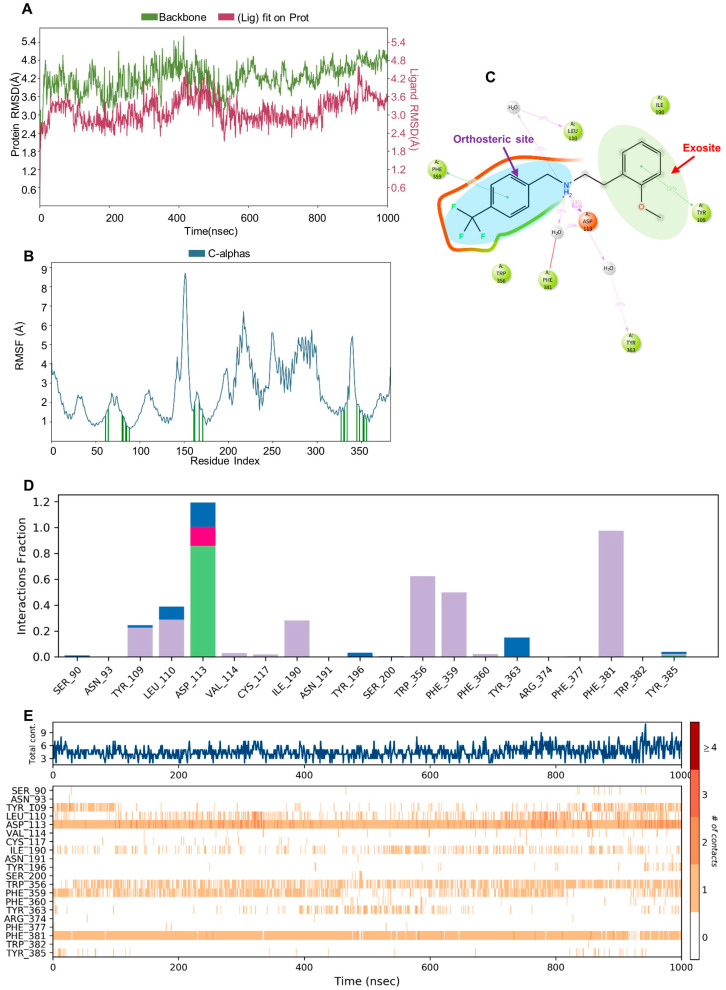
MD simulation of **SY-17** and α_2A_-AR complex. (**A**) RMSD plot of α_2A_-AR with ligand **SY-17**. The ligand was aligned to the protein and then calculated RMSD; (**B**) RMSF plot of α_2A_-AR, the green lines illustrated certain residues which contacted with ligand; (**C**) fraction of simulation time of specific residue interactions during 1000 ns simulation shown with L-P plot. The displayed residue interacted with the ligand for at least 10% of the simulation time; (**D**) interaction fractions of α_2A_-AR active residues with **SY-17** (hydrogen bonds are shown with green bars; ionic and water bridges are shown with pink and blue color bars; hydrophobic are shown with purple); (**E**) plots of protein–ligand contacts and interactions during 1000 ns simulation.

To be more specific, MD simulations were also carried out to evaluate the stability of interactions and motions for **SY-15** and **SY-17** with α_2A_-AR docked complexes (Figure 6 and Figure S3). The RMSD was calculated over the whole 1000 ns trajectory to estimate conformational differences between the original structure and subsequent snapshots throughout the simulation. As shown in Figure 6A, the RMSD values for α_2A_-AR reached equilibrium after approximately 0.6 μs of simulation time and followed convergence to the rest of the simulation, indicating that the system had stabilized and the simulation could be terminated. The average RMSD values for ligands and proteins were relatively small, demonstrating that the complex was stable throughout the trajectory. Subsequently, the RMSF method was used to qualitatively analyze the fluctuating information for each amino acid residue of α_2A_-AR. As depicted in Figure 6B, since α_2A_-AR is a multiple transmembrane protein, the secondary structure of the lower fluctuating residues was α-helix, and these residues were in the seven transmembrane segments of the receptor and were stabilized by phospholipid membranes. Furthermore, the higher fluctuating residues were typically located in the loop regions of the extra- or intracellular segments, which conferred greater flexibility. The green lines illustrated certain residues which contacted with ligand, including Trp356, Phe359, and Phe381 located in the orthosteric site, as well as Ser90 and Asn93 in the exosite.

To identify specific interactions including hydrophobic interactions and hydrogen bonds, the 2D ligand interaction diagram was generated between the ligand and binding site of the protein. As shown in Figure 6C,D, **SY-17** continuously interacted with Asp113 in TM3 via a hydrogen bond and salt bridge within the orthosteric site. The residues Trp356, Phe359, and Phe381 also contributed to the binding of **SY-17** via hydrophobic interactions. Moreover, **SY-17** formed additional interactions with Ser90, Asn93, Trp109, and Leu110 in the exosite.

Additionally, the blue-colored plot indicated that an average of 4–5 residues were in contact with **SY-17** during the whole 1000 ns simulation. Residues involved in more than one interaction with **SY-17** were colored with darker shades of orange (Figure 6E). Asp113 and Phe381, which are shown with orange shades, were maintained throughout the simulation, suggesting the specific and consistent binding of **SY-17** to α_2A_-AR active site.

The binding free energy was calculated by MM/GBSA and decomposed into individual residues. Total free energy (ΔG) of **SY-15** and **SY-17** was consistently lower than −45 kcal/mol during the MD simulations (Figure S4). Generally, there exists a positive correlation between the binding free energy of a compound and its affinity towards the receptor. Through MD simulations, it was observed that despite **SY-17** exhibiting a lower binding free energy (−71.97 kcal/mol) compared to **SY-15** (−45.93 kcal/mol), its efficacy in reducing intracellular cAMP levels was inferior to that of **SY-15**. This phenomenon may be attributed to the fact that, in the context of agonists, the affinity between the compound and receptor does not directly correlate with the receptor’s activation efficiency, because the agonist will induce the conformational changes for the receptor. Consequently, even if some compounds demonstrate high affinity, they may not activate the receptor if they cannot induce the receptor to produce a specific conformation.

**Figure 7:**
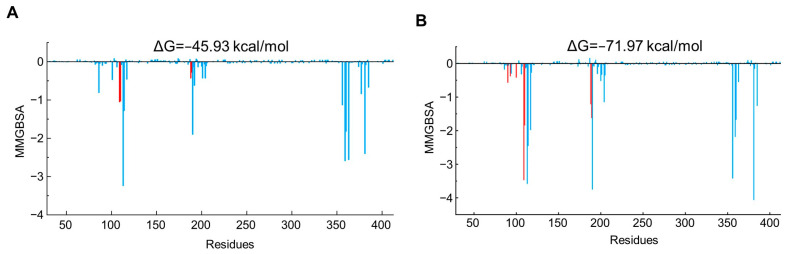
(**A**) Decomposed binding free energy of **SY-15** (residues located in the orthosteric site were colored blue, while in exosites were red); (**B**) binding free energy of **SY-17**.

The most significant contribution to ΔG for **SY-17** mainly came from Van der Waals interactions, in contrast to DMED, which relied on an electrostatic interaction with Asp113. Residues Tyr109, Leu110, Cys188, and Glu189 located in exosite also interacted with **SY-15** and **SY-17** (Figure 7A,B). Collectively, these data proved that **SY-15** and **SY-17** could form stable protein–ligand complexes with α_2A_-AR and both act as bitopic α_2A_-AR agonists by simultaneously occupying the orthosteric site and exosite.

**Figure 8:**
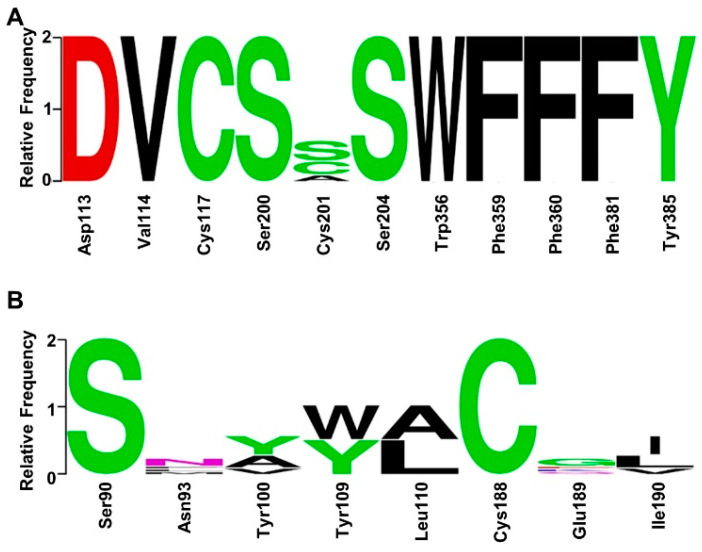
(**A**) α-AR receptor family sequence conservation in orthosteric sites; (**B**) sequence conservation of exosites.

Compared with traditional orthosteric ligands, bitopic ligands demonstrated a higher affinity and selectivity from their additional interactions with less-conserved allosteric sites across the GPCR family [9]. Lastly, we evaluated the sequence conservation of orthosteric sites and exosites for all α-ARs, including α_1A_, α_1B_, α_1D_, α_2A_, and α_2B_, as well as α_2C_ subtypes. As shown in Figure 8A,B and Figure S5, the orthosteric site exhibited high conservation among these subtypes, while Asn93, Tyr100, Glu189, and Ile190 in the exosite were significantly different from the homologous residues of other receptors. Thus, bitopic agonists which occupied the orthosteric site and exosite may simultaneously exhibit the potential for increased subtype selectivity.

## 3. Materials and Methods

### 3.1. Protein Preparation

The crystal structure of α_2A_-AR in complex with antagonist RSC (PDB ID:6kux) was retrieved from the Protein Data Bank [8,20]. The antagonist RSC was removed using PyMOL 2.5.5, and then α_2A_-AR was exported to a single PDB file [21,22]. Subsequently, the protein was added to hydrogen and water was removed by the Protein Preparation Wizard in Maestro, and the missing side chains and loops were filled using Prime [23].

### 3.2. Molecular Dynamics Simulation

MD simulations of the receptor alone and in complex with candidate compounds were respectively conducted using the Desmond module of the Schrodinger suite. The ligands-α_2A_-AR complex structures were obtained from docking studies conducted with Maestro. Receptor or receptor–ligand complex files were submitted to the Desmond employing the OPLS_2005 force field with a TIP3P solvent model [24]; each was inserted in the phosphatidylcholine (POPC) lipid membrane. Firstly, proteins or protein–ligand complexes were prepared with the system builder panel, which added periodic boundary conditions and defined orthorhombic boxes. An appropriate amount of Na^+^ or Cl^−^ ions was added to the system to neutralize charges, and the sodium chloride molecules were added to reach the physiological concentration of 0.15 M. The system was also translated into a local energy minimum before simulations. The prepared system underwent 1500 ns MD simulations with parameters of a 300 K reference temperature and 1.01 bar pressure with a time step of 2 fs. The Nosé–Hoover temperature coupling method and the Martyna–Tobias–Klein barostat method with applied isotropic coupling algorithm were used to maintain pressure and temperature parameters during simulations [25,26,27]. Particle Mesh Ewald (PME) and the SHAKE algorithm were employed to calculate long-range electrostatic interactions and constrain covalent bonds [28,29,30]. Root mean square deviations (RMSD), root mean square fluctuations (RMSF), hydrogen bond interactions, and ligand–protein contacts were analyzed in the final generated trajectory report. MM/GBSA binding free energy during the MD simulation was calculated using the thermal_mmgbsa.py script and subsequently decomposed using the breakdown_MMGBSA_by_residue.py script.

For receptors without ligands, 10 representative conformations were extracted from the trajectory by a clustering tool. The clustering analysis was performed using hierarchical clustering with average linkage, and the RMSD of the backbone served as the structural similarity metric.

### 3.3. Virtual Screening

The Chemdiv compound library was pre-filtered according to Lipinski’s rules. All compounds were prepared using LigPrep; the stereoisomers, ionization states at pH 7.0 ± 2.0, and tautomers were generated by Epik with the OPLS3 force field [31,32,33]. Grids for the binding site were defined using 10 clusters from MD simulation for the apo-protein system and one conformation of the crystal structure through Receptor Grid Generation. Ligands were docked into a total of 11 grids by Glide HTVS and SP with default parameters [32,34]. Ten percent of the top-scoring molecules in HTVS were further ranked by SP. Ligands that exhibited the higher average scores for each conformation in SP docking were evaluated by the binding free energy. These maintained molecules were then clustered by structural similarity using Maestro.

### 3.4. MM/GBSA Binding Free Energy (ΔG)

The binding free energy (ΔG) of the maintained molecules to α_2A_-AR was calculated using the molecular mechanics/generalized born surface area (MM/GBSA) method, in which ΔG is defined as the following:

ΔG = ΔE_vdW_ + ΔE_ele_ + ΔG_pol_ + ΔG_nonpol_ (1)

where ΔE_vdW_ represents the energy contribution of van der Waals interactions, and ΔE_ele_ is the energy contribution of electrostatic interactions in the gas phase. ΔG_pol_ and ΔG_nonpol_ are the energy contribution of the polar and nonpolar solvation, respectively [35].

### 3.5. Visual Inspection Screening

In this manual screening stage, molecules with MM/GBSA scores lower of −40 kcal/mol were selected through careful and comprehensive consideration of key interactions and binding poses with α_2A_-AR. Protein–ligand interactions and binding poses were analyzed in Maestro. Following the result of cluster analysis, 2–3 compounds were selected as candidate molecules from each cluster for further investigation.

### 3.6. Protein Kinase A (PKA) Redistribution Assay In Vitro

CHO-PKA-cat α_2A_-AR cells were cultured in 96-cell plates and pre-treated with forskolin (10 μM) for 15 min, then incubated with candidate compounds. Cells were fixed and the formation of cytoplasmic spots were quantitatively measured using the Cellomics Array Scan VTI Reader and the Spot DetectorV3 BioApplication of a high-throughput screening assay. Activity was calculated as follows:

Activity (%) = (test signal − negative control signal)/(positive control signal − negative control signal) × 100%

For positive controls, cells were pre-treated with forskolin for 15 min, then treated with the agonist DMED. Negative control cells were treated with 0.25% DMSO and forskolin. Furthermore, the antagonistic activity of compounds was tested in CHO-α_2A_-PKAcatEGFP cells pre-treated with forskolin (10 μM) and DMED (10 μM).

### 3.7. cAMP Assay In Vitro

HEK293 cells were co-transfected with the pGloSensor-22F cAMP plasmid (Promega, Madison, WI, USA, E1171) and pCMV6 Entry-Flag-α_2A_-AR, following the procedure of GloSensor cAMP biosensor (Promega) manufacturer’s protocols. On the next day, cells were seeded in white 96-well plates. The medium was replaced with 90 μL of fresh DMEM containing 2% *v*/*v* GloSensor cAMP reagent (Promega, E1290), then incubated for 60 min at 37 °C. The baseline signal was initially recorded before the cells were treated with 10^−12^–10^−4^ M candidate compounds for 10 min and stimulated by forskolin (10 μM) for 15 min. The cAMP accumulations induced by DMED or tested compounds were also measured in co-transfected HEK293 cells. In each experiment, cells transfected with pcDNA3.1 myc/hisB served as the negative control, while cells stimulated with 10 μM forskolin acted as the positive control [36]. Signals were detected using the Victor 2D Instrument (PerkinElmer) at 675 nm. Levels of cAMP were calculated by the following method:

cAMP(%) = (signal after treatment − baseline signal)/baseline signal × 100%

## 4. Conclusions

The α_2A_-AR is a critical target for sedative and analgesic drugs. However, the orthosteric site, which is highly conserved among it and other subtypes, results in low selectivity and unavoidable side effects of imidazole-ring α_2A_-AR ligands. In the present study, the ensemble-based screening strategy integrating MD simulation with molecular docking was employed to identify potential α_2A_-AR ligands with novel scaffolds. Cell-culture assays validated that the compounds **SY-15** and **SY-17** dose-dependently reduced the levels of intracellular cAMP. Molecular docking revealed that the binding modes of **SY-15** and **SY-17** were different from the traditional imidazole ring compound DMED; these two agonists also interacted with residues in an exosite, which was located above the orthosteric site in the extracellular domain of α_2A_-AR. MD simulations further indicated that both **SY-15** and **SY-17** can occupy the orthosite and exosite simultaneously. By analyzing the sequence conservation of the binding site, we found that compounds occupying both the orthosteric site and exosite may exhibit higher isotype selectivity, which provides the theoretical basis for the subsequent discovery of novel highly selective bitopic agonists. In summary, **SY-15** and **SY-17** can be further studied as bitopic agonists for α_2A_-AR, and the predicted model of **SY-15** and **SY-17** in complex with α_2A_-AR may serve as an important starting point for the optimization of high-selectivity bitopic leads and provide new avenues for the development of sedative and analgesic drugs.

## Figures and Tables

**Table 1:** Mean docking score and binding free energy for 20 candidate compounds.

No.	Compound Name	Docking Score (kcal/mol)	MM/GBSA dG Bind (kcal/mol)
1	**SY-1**	−8.442	−60.09
2	**SY-2**	−8.515	−56.44
3	**SY-3**	−8.592	−37.35
4	**SY-4**	−9.945	−69.46
5	**SY-5**	−9.997	−64.42
6	**SY-6**	−10.191	−81.83
7	**SY-7**	−9.097	−77.21
8	**SY-8**	−9.020	−70.94
9	**SY-9**	−9.002	−58.34
10	**SY-10**	−8.690	−61.14
11	**SY-11**	−9.169	−56.19
12	**SY-12**	−9.904	−66.14
13	**SY-13**	−9.575	−77.31
14	**SY-14**	−8.896	−72.72
15	**SY-15**	−9.872	−50.98
16	**SY-16**	−9.836	−53.23
17	**SY-17**	−9.450	−54.95
18	**SY-18**	−9.447	−32.94
19	**SY-19**	−9.248	−53.99
20	**SY-20**	−9.009	−40.57

## Data Availability

Data are contained within the article and Supplementary Materials.

## Supplementary Materials

The following supporting information can be downloaded at https://www.mdpi.com/article/10.3390/molecules29051097/s1. Figure S1. α_2A_-AR receptor and lipid bilayer membrane model used in the molecular dynamic simulations. Figure S2. The chemical structure of candidate molecules. Figure S3. MD simulation of **SY-15** and α_2A_-AR complex. Figure S4. Constituent parts of binding free energy for **SY-15** during the MD simulation; and constituent parts of binding free energy for **SY-17**. Figure S5. Multiple sequences alignment of orthosteric sites for α-AR receptor family, and multiple sequences alignment of exosite for α-AR receptor family. Table S1. The ADME/T properties of the candidate compounds for in vitro assay.
