# Supplementary material for: Novel Scaffold Agonists of the α2A Adrenergic Receptor Identified via Ensemble-Based Strategy
Source: Molecules. 2024 Feb 29;29(5):1097. doi: 10.3390/molecules29051097 (PMC10934406; doi:10.3390/molecules29051097)
Supplement: Supplementary file 1 [file molecules-29-01097-s001.zip › molecules-2886653-supplementary.pdf]

Supplementary Materials

1. Supplementary Figures

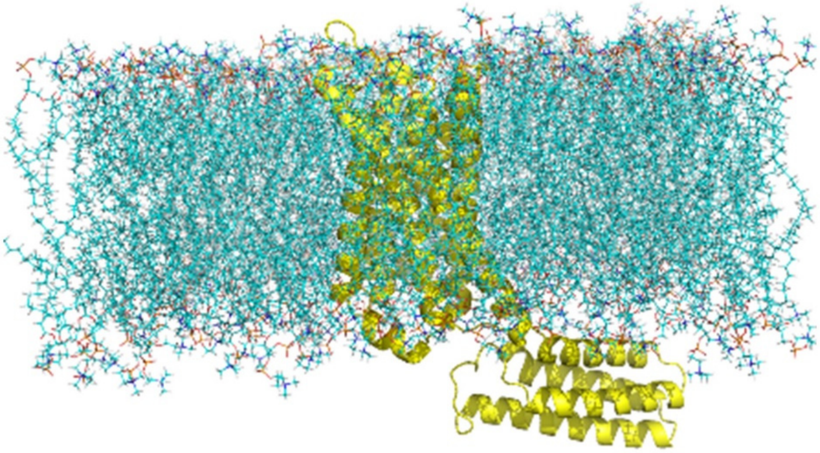

Figure S1. α_{2A}-AR receptor and lipid bilayer membrane model used in the molecular dynamic simulations.

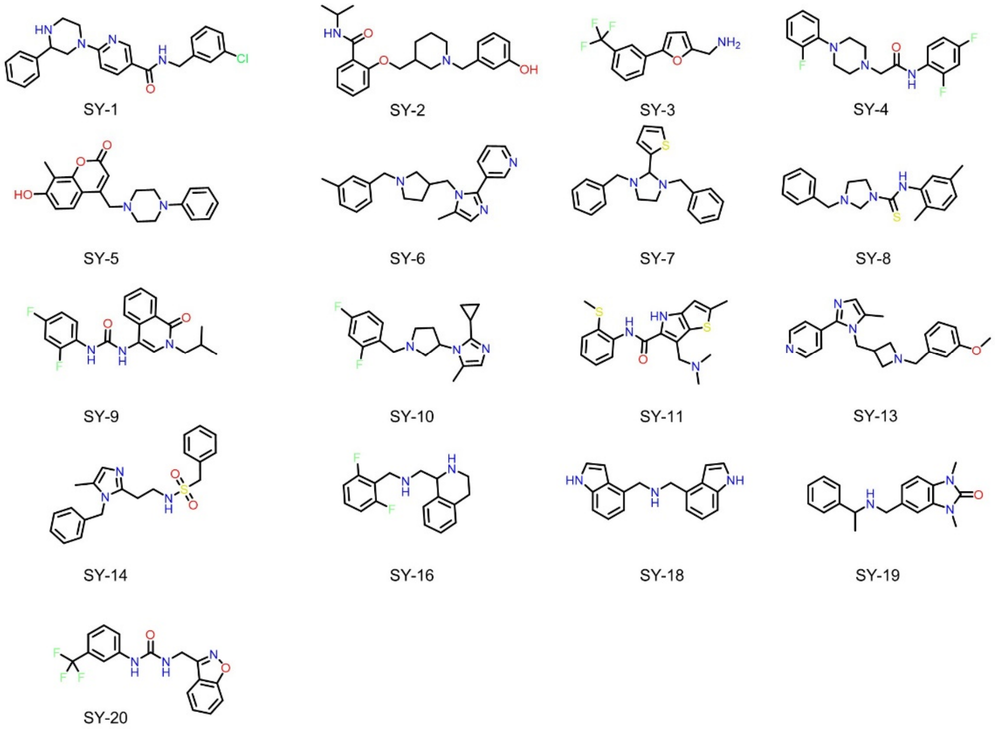

Figure S2. The chemical structure of candidate molecules.

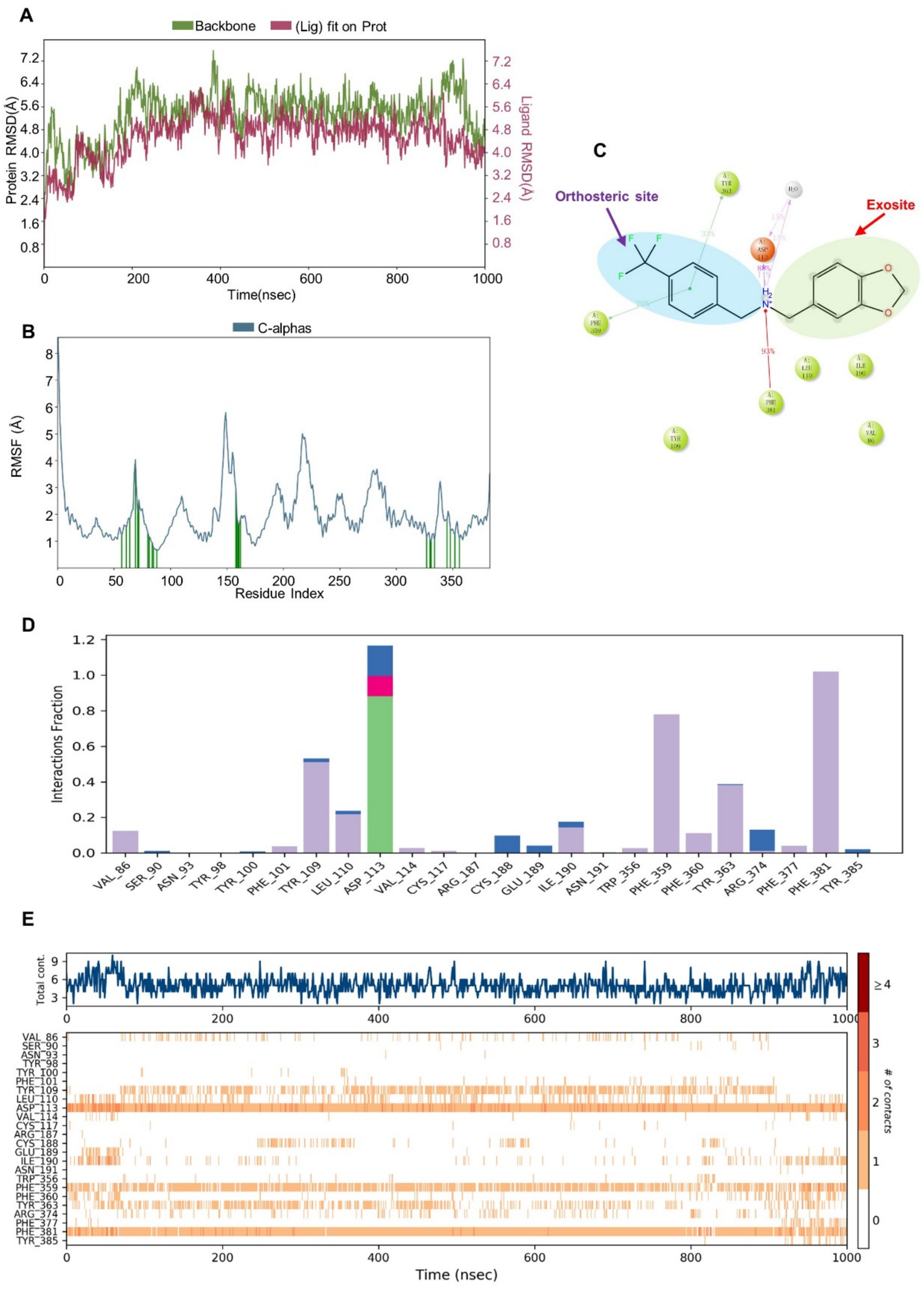

Figure S3. MD simulation of **SY-15** and α_{2A}-AR complex. (**A**) The RMSD plot of α_{2A}-AR with ligand **SY-15**; (**B**) The RMSF plot of α_{2A}-AR; (**C**) The percentage of simulation time of residue interactions during 1000 ns simulation shown with L-P plot; (**D**) The interaction fractions α_{2A}-AR active residues with ligand **SY-15**; (**E**) The plots of protein-ligand contacts and interactions during 1000 ns simulation;

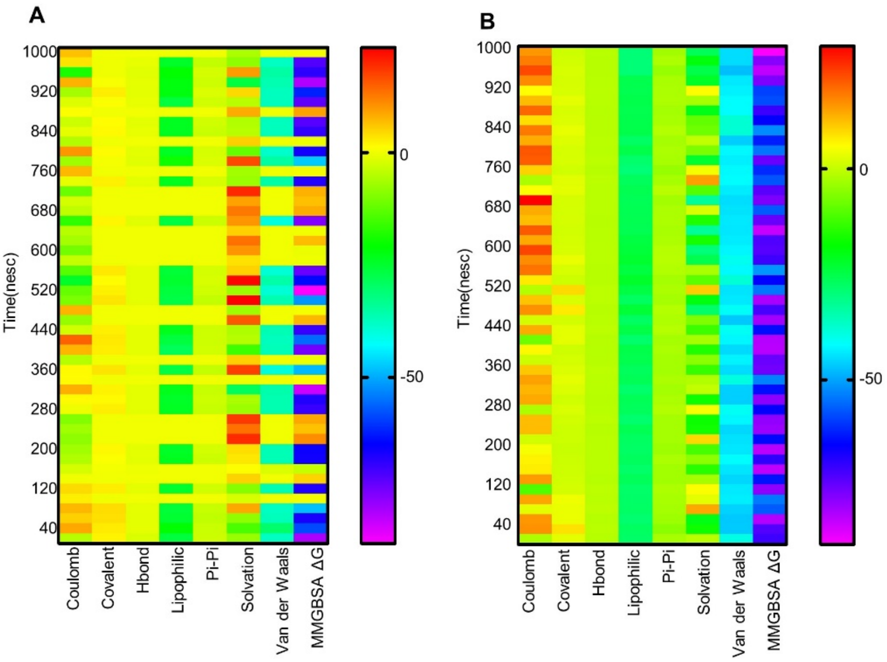

Figure S4. (**A**) Constituent parts of bind free energy for **SY-15** during the MD simulation; (**B**) Constituent parts of bind free energy for **SY-17**.

A

P08913	ADA2A	D	V	C	S	C	S	W	F	F	F	Y
P18089	ADA2B	D	V	C	S	S	S	W	F	F	F	Y
P18825	ADA2C	D	V	C	S	C	S	W	F	F	F	Y
P35348	ADA1A	D	V	C	S	A	S	W	F	F	F	Y
P35368	ADA1B	D	V	C	S	S	S	W	F	F	F	Y
P25100	ADA1D	D	V	C	S	S	S	W	F	F	F	Y

B

P08913	ADA2A	S	N	Y	Y	L	C	E	I
P18089	ADA2B	S	N	Y	Y	L	C	K	L
P18825	ADA2C	S	N	Y	Y	L	C	G	L
P35348	ADA1A	S	F	A	W	A	C	Q	I
P35368	ADA1B	S	L	V	W	A	C	G	V
P25100	ADA1D	S	M	A	W	A	C	G	I

Figure S5. (**A**) Multiple sequences alignment of orthosteric sites for α-AR receptor family; (**B**) Multiple sequences alignment of exosite for α-AR receptor family.

2. Supplementary Tables

Table S1. The ADME/T properties of the candidate compounds for in vitro assay

Compound Name	IDNUMBER	PSA	SASA	logP	WPSA	FOSA	QPlogHERG	rotatable bonds	FISA	HumanOral Absorption	CNS	QPlogBB	RuleOfFive	donorHB	accptHB	mol MW
**SY-1**	P801-0655	63.73	750.87	4.20	71.57	162.45	−8.04	6	79.72	1	1	0.07	0	2	5.5	406.91
**SY-2**	M735-1116	65.00	716.06	2.49	0.00	342.89	−6.76	9	87.75	3	1	−0.42	0	2	6	382.50
**SY-3**	8019-8601	34.04	465.04	3.29	117.63	58.53	−5.61	3	61.10	3	2	0.58	0	2	1.5	241.21
**SY-4**	K940-1464	43.63	623.47	3.20	105.25	163.18	−6.72	5	45.18	3	2	0.59	0	1	5.5	349.36
**SY-5**	Y041-4036	67.45	652.16	3.08	0.00	245.44	−6.71	4	117.07	3	1	−0.41	0	1	6.25	350.42
**SY-6**	S779-1061	34.93	656.05	3.05	0.00	275.67	−6.58	5	58.10	3	1	0.16	0	0	5	346.47
**SY-7**	8007-8950	10.99	615.44	3.39	26.06	119.64	−7.77	5	8.06	3	2	1.11	0	0	4	334.48
**SY-8**	K788-3789	26.30	639.59	5.27	48.50	289.08	−6.60	5	16.65	3	2	0.73	0	1	4.5	325.47
**SY-9**	E942-0016	68.95	710.18	2.93	89.10	187.20	−5.53	6	89.43	3	0	−0.47	0	2	5	371.39
**SY-10**	S694-0761	21.01	594.29	3.59	70.81	318.20	−5.75	4	20.85	3	2	0.82	0	0	3.5	317.38
**SY-11**	M372-1199	51.43	642.78	3.82	63.61	328.19	−6.09	6	38.04	3	1	0.45	0	2	5	359.50
**SY-12**	P801-0725	61.30	737.95	3.51	0.00	251.75	−7.77	6	62.80	3	1	0.10	0	2	5.5	386.50
**SY-13**	S696-1461	42.75	712.25	1.86	0.00	292.19	−6.70	6	57.44	1	0	−0.36	1	0	3.75	348.45
**SY-14**	T161-0947	66.47	681.71	1.78	0.00	174.72	−6.45	8	101.54	3	-1	−0.94	0	1	6	369.48
**SY-15**	4964-4916	32.40	507.64	3.99	117.34	154.68	−5.22	5	16.75	3	2	0.87	0	1	3	309.29
**SY-16**	8013-2958	23.82	537.49	2.87	63.58	164.58	−6.63	4	21.51	3	2	1.08	0	2	3	288.34
**SY-17**	4964-4708	17.98	572.37	4.53	117.11	204.32	−5.99	7	11.55	3	2	0.78	0	1	2.25	309.33
**SY-18**	8019-9725	40.95	537.20	3.17	0.00	48.26	−6.53	4	74.70	3	1	0.04	0	3	1.5	275.35
**SY-19**	D215-0599	46.07	596.22	2.93	0.00	266.21	−6.28	4	66.44	3	1	0.08	0	1	3.5	295.38
**SY-20**	E567-0008	74.01	574.01	3.34	114.04	42.82	−4.40	6	104.58	3	0	−0.35	0	2	3.5	335.29
